# The golden ratio in the pulmonary circulation in patients with heart failure and cardiogenic shock

**DOI:** 10.14814/phy2.70287

**Published:** 2025-03-28

**Authors:** Hoong Sern Lim, Ivan H. W. Yim

**Affiliations:** ^1^ University Hospitals Birmingham NHS Foundation Trust Birmingham UK; ^2^ Institute of Cardiovascular Sciences University of Birmingham Birmingham UK

**Keywords:** cardiogenic shock, heart failure, hemodynamics, pulmonary hypertension

## Abstract

The consistent relationship between pulmonary artery systolic (PASP), diastolic (PADP), mean (mPAP), and pulse (PP) pressures has led to the proposal of the golden ratio (Φ) hypothesis. This study tested the Φ hypothesis in the pulmonary circulation of patients with advanced heart failure (HF) and cardiogenic shock (CS). PASP:mPAP, mPAP:PADP, and PP:mPAP ratios were evaluated in 20 patients with advanced HF (high‐fidelity measurements) and 93 patients with CS (fluid‐filled system). Twelve of 20 patients with advanced HF had PASP:mPAP and mPAP:PADP ratios from high‐fidelity measurements that were consistent with the Φ hypothesis. The eight patients with low PASP:mPAP and mPAP:PADP ratios had lower PP: mPAP ratio (0.72 (0.71–0.73) vs. 0.94 (0.92–0.99), *p* = 0.002). In patients with CS, 76 patients (82%) had PASP:mPAP and mPAP:PADP ratios that were consistent with the Φ hypothesis. The 17 patients with CS and “low ratios” also had lower PP:mPAP (0.53 (0.48–0.57) vs. 0.74 (0.68–0.87), *p* < 0.001). Lower PP:mPAP ratio was related to higher filling pressures and lower cardiac power output. The pulmonary circulation deviated from the Φ hypothesis in patients with more severe HF and CS. Low PP:mPAP ratio identifies patients with HF and CS with more severe hemodynamic compromise.

## Graphical abstract

Stroke volume is a determinant of both mPAP (left wireframe plot) and PP (right surface plot). Mean pulmonary artery pressure changes with SV with a coefficient of (PVR/*t*, where *t* = cardiac interval). The mPAP plot is based on a PVR of 4.2WU (or 0.252 mmHg/mL.s) at cardiac intervals from 400 to 1000 ms and PAWP of 29 mmHg. Pulmonary artery PP changes with SV with a coefficient of 1/PAC. As a result, PP falls more steeply with reduction in SV than mPAP, resulting in lower PP: mPAP ratio with reduction in SV. For example, at PVR of 4.2WU, PAC of 1.2 mL/mmHg, SV of 60 mL, HR of 100 bpm, PAWP of 29 mmHg, the PP and mPAP are 50 mmHg and 54 mmHg, respectively (PP: mPAP = 0.93). At the same PVR, PAC, HR, and PAWP, but SV of 30 mL, the PP and mPAP are 25 mmHg and 42 mmHg (PP: mPAP = 0.60). Pulmonary artery PP that is significantly lower than mPAP deviates from the Φ hypothesis.

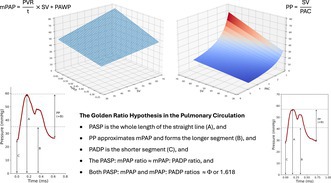

## 1 INTRODUCTION

Clinicians have long observed the consistent and predictable relationships between different components of the pulmonary artery (PA) pressure in individuals without pulmonary hypertension (PH) and in patients with PH due to various etiologies (Syyed et al., 2008). The relationship between PA systolic pressure (PASP) and mean PA pressure (mPAP) was such that some have suggested that these components of PA pressures may even be “interchangeable” (Chemla et al., 2015). Two observations were especially notable: the approximation of PA pulse pressure (PP) to mPAP, and the remarkably reproducible relationship between PASP and mPAP under various conditions and pathologies (Vanden Eynden et al., 2016). The basis for these two observations has been attributed to the longitudinal distribution of PA compliance into smaller pulmonary vessels (Presson Jr et al., 1988), an anatomical feature that is distinct from the systemic circulation. These two observations have also provided the rationale for the golden ratio hypothesis (Φ hypothesis) in the pulmonary circulation. Chemla et al. (2019) building on these observations, reported that the PA pressures demonstrated features that were consistent with the Φ hypothesis in patients with and without PH.

The golden ratio refers to a specific property: for a straight line that is divided into two unequal segments, the ratio of the whole length of the line (A) divided by the long segment of the line (B) is the same as the ratio of the long segment divided by the short segment of the line (C), and both ratios approximate the irrational number phi, Φ of 1.618. Several investigators have observed the golden ratio, or Φ in the cardiovascular system, including the coronary circulation and the human heart (Ulmer et al., 2009).

The golden ratio hypothesis has not been examined in patients with advanced heart failure (HF) and PH due to left heart disease (PHLHD) and under conditions of more extreme hemodynamic distress such as cardiogenic shock (CS). This study tested the hypotheses that (i) the Φ hypothesis in the pulmonary circulation fails in patients with advanced HF and CS; and (ii) deviation from the Φ hypothesis in the pulmonary circulation is associated with more severe systemic hemodynamic compromise in patients with HF and CS.

## 2 METHODS

This study consisted of two cohorts of patients—the HF cohort and CS cohort. The first cohort included 20 patients with advanced HF who underwent right heart catheterization as assessment for heart transplantation—the HF cohort. A combined dual‐tipped pressure and Doppler flow sensor wire (Combowire; Philips Volcano, sampling rate 200 Hz) was inserted via the catheter to acquire high‐fidelity pressure measurements and Doppler flow data as previously described by Yim et al Yim, Parker, et al. (2024). The high‐fidelity pressure data were exported for post‐processing. Mean PA pressure was calculated as the area under the pressure curve (trapezoidal method, Scipy, Python v3.13.1). This study was approved by the Health Research Authority and Health Care Research Wales ethics committee (reference 20/WM/0022), with written consent from all participants.

The second cohort included 93 consecutive patients with cardiogenic shock from April 2016 to April 2024 who underwent pulmonary artery catheterization using standard fluid‐filled manometry, as part of an ongoing institutional project to protocolize cardiogenic shock care (Lim, 2022)—the CS cohort. Patients who were deteriorating rapidly and too unstable for pulmonary artery catheterization, ongoing cardiac arrest, and concurrent obstructive shock (e.g., tamponade) were excluded. Right heart catheterization was performed in the standard manner with a balloon‐tipped, fluid‐filled 7.5F Swan Ganz catheter via the jugular vein. Zero reference was set at the mid‐chest and measurements were averaged over the respiratory cycle. Hemodynamic traces were reviewed (IY) and the computer‐measured mean was taken used for mPAP. Cardiac output was measured by the thermodilution technique and three values differing by <10% were averaged. All hemodynamic data were collected at the time of insertion. This study has institutional approval (CARMS‐17781) and patient consent was waived. Due to the anonymization of the data, the age was simply reported as >50 years or ≤50 years, instead of mean and standard deviation.

### 2.1 Theoretical considerations

The golden ratio can be expressed algebraically as: A/B = B/C = Φ = 1.618. Solving this equation, with A = B + C, so (B + C)/C = B/C, reveals the remarkable “self‐defining” property of the Golden ratio: Φ = 1 + 1/Φ. Multiplying by Φ (i.e., exponent), it is evident that each power is the sum of the two powers before it, which is analogous to the Fibonacci Sequence. The ratio of the number on the Fibonacci Sequence to the sum of the two numbers before it on the Fibonacci Sequence also approximates Φ. As Φ = 1 + 1/Φ, so 1/Φ = Φ – 1 = 0.618.

Chemla et al. noted that 1/Φ of 0.618 is close to the coefficient or slope of the empirical formula for deriving mean PA pressure from PASP (Chemla et al., 2004):

mPAP=0.61×PASP+2

This empirical formula has been studied over a range of PA pressures in different pathological conditions and patient characteristics. Omitting the “small” intercept, the ratio of PASP to mPAP approximates Φ:

mPAP=0.61×PASP,or

$$\frac{\text{PASP}}{\text{mPAP}}\approx 1.62$$

**FIGURE 1:**
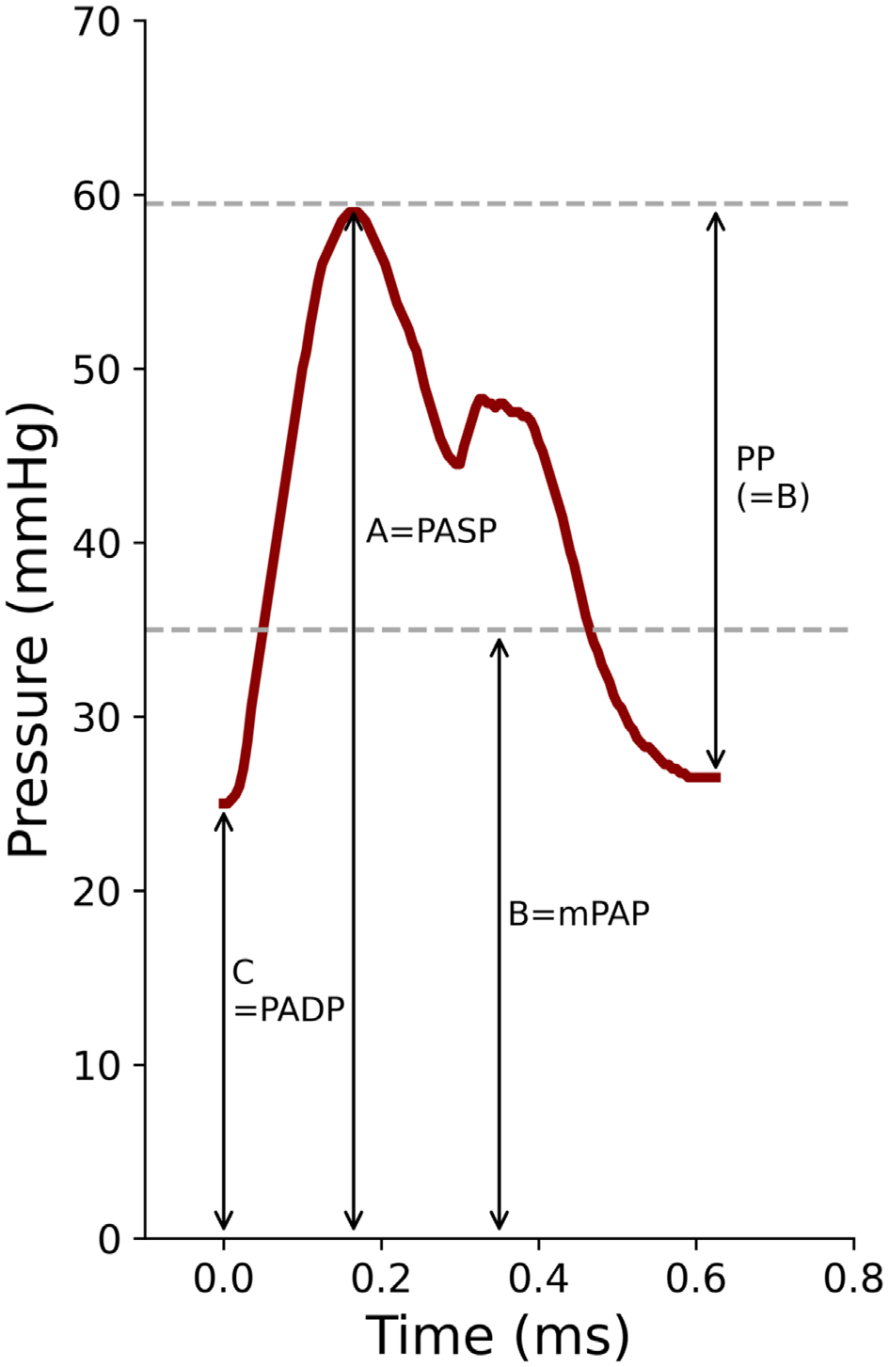
High‐fidelity pressure recording from the PA. Mean PA pressure was derived from the integration of the area under the pressure trace. To fulfill the Φ hypothesis, A/B = B/C = 1.618 or Φ. The implication is that PA PP should approximate mPAP (lower horizontal dashed line).

There were two additional observations of note. In a cohort of patients with pulmonary hypertension, the ratio of mPAP to PA diastolic pressure (PADP) was close to Φ, especially if a 2% error margin is assumed; and the PA pulse pressure (PP) was close to the mPAP (PP: mPAP was 0.95) (Chemla et al., 2019). Thus, the Golden Ratio hypothesis is fulfilled if:

1. PASP is the whole length of the straight line (A), and
2. PP approximates mPAP and forms the longer segment (B), and
3. PADP is the shorter segment (C), and
4. The PASP: mPAP ratio ≈ mPAP: PADP ratio, and
5. Both PASP: mPAP and mPAP: PADP ratios ≈ Φ or 1.618 (Figure 1).

Based on these results, Φ has been proposed as the “constant scaling factor” that determines the relationship between systolic, diastolic, and mean pressures in the pulmonary circulation.

### 2.2 Testing the golden ratio hypothesis

The proportionality between PASP, PADP, and mPAP is the basis for the Φ hypothesis, that is, (i) the ratios of PASP: mPAP and mPAP: PADP approximate Φ; and (ii) pulmonary artery PP approximates mPAP such that PP: mPAP approaches 1.0 (Figure 1).

(i) The PASP: mPAP and mPAP: PADP ratios

In practice, PASP: mPAP and mPAP: PADP ratios that are higher or lower than Φ may nonetheless be consistent with the Φ hypothesis due to the inherent error in pulmonary artery pressure measurements. A 5% and 10% error limit for high‐fidelity and fluid‐filled catheters (Falsetti et al., 1974) were used for this study, which corresponds to a range of 1.46–1.79 and 1.32–1.97, respectively, for both PASP: mPAP and mPAP: PADP ratios.

(ii) The PP: mPAP ratio

Previous studies have reported mean/ median PP: mPAP ratios of 0.88 (0.23) and 0.95 (interquartile range, IQR 0.82–1.10) in patients without pulmonary hypertension and in patients with pulmonary hypertension due to various pathologies (Chemla et al., 2009; Weatherald et al., 2018), which were consistent with the Φ hypothesis. On review of the hemodynamic data from 10 studies (summarized in Table S1), the median PP: mPAP ratio was 0.89 (IQR 0.79–0.96), which was comparable to previous studies. Using the convention of 1.5 × IQR to define outliers, the upper and lower limits of PP: mPAP based on these 10 studies of patients with HF and PHLHD were 0.612 and 1.228, that is, PP: mPAP ratios within these limits were considered to be consistent with published data in patients with HF.

Although the proportionality of the PASP:mPAP, mPAP:PADP, and PP:mPAP ratios are features of the golden ratio, we focused specifically on the PP:mPAP ratio because (i) it is too cumbersome to present data for all three ratios; (ii) the normal PP:mPAP ratio of 1.0 is easier to work with compared to the ratios PASP:mPAP or mPAP:PADP of 1.618; and (iii) the proportionality of PP‐to‐mPAP has been a central feature of previous physiological evaluation into the pulmonary circulation (Saouti, Westerhof, Helderman, et al., 2010), and (iv) the PP:mPAP ratio has already been described previously in cardiogenic shock (Lim, 2024).

### 2.3 Relationship with systemic hemodynamic parameters

The second part of this study will examine the relationship between deviation from Φ and the severity of systemic hemodynamic derangements. The association with cardiac power output index (CPOI) was specifically evaluated due to the well‐established association with mortality in CS (Baldetti et al., 2022). The calculation of CPOI included right atrial pressure as previously described (Lim, 2020):

CPOI=MAP−RAP×CI451,

where MAP, mean arterial pressure; RAP, right atrial pressure, and CI, cardiac index.

Pulmonary artery capacitance (PAC) is usually calculated as (Al‐Naamani et al., 2015):

PAC=SV/PP

This equation for PAC has inherent inaccuracies as it does not take into consideration the continuous outflow from the pulmonary circulation.

### 2.4 Statistical analysis

Normality was assessed with the Kolmogorov–Smirnov test. Continuous variables were expressed as median (interquartile range, IQR) for non‐normally distributed or mean ± SD for normally distributed variables. Comparisons were performed using Mann–Whitney's (independent samples) tests and *t*‐tests, respectively. The fractional PASP (i.e., PASP: mPAP), 1/fractional PADP (i.e., mPAP: PADP), and proportional pulmonary artery pulse pressure (i.e., PP: mPAP) were calculated as simple ratios. All tests were two‐sided, with a *p* < 0.05 considered statistically significant. All analyses were performed on Python (v3.13.1).

## 3 RESULTS

### 3.1 Advanced HF cohort

**FIGURE 2:**
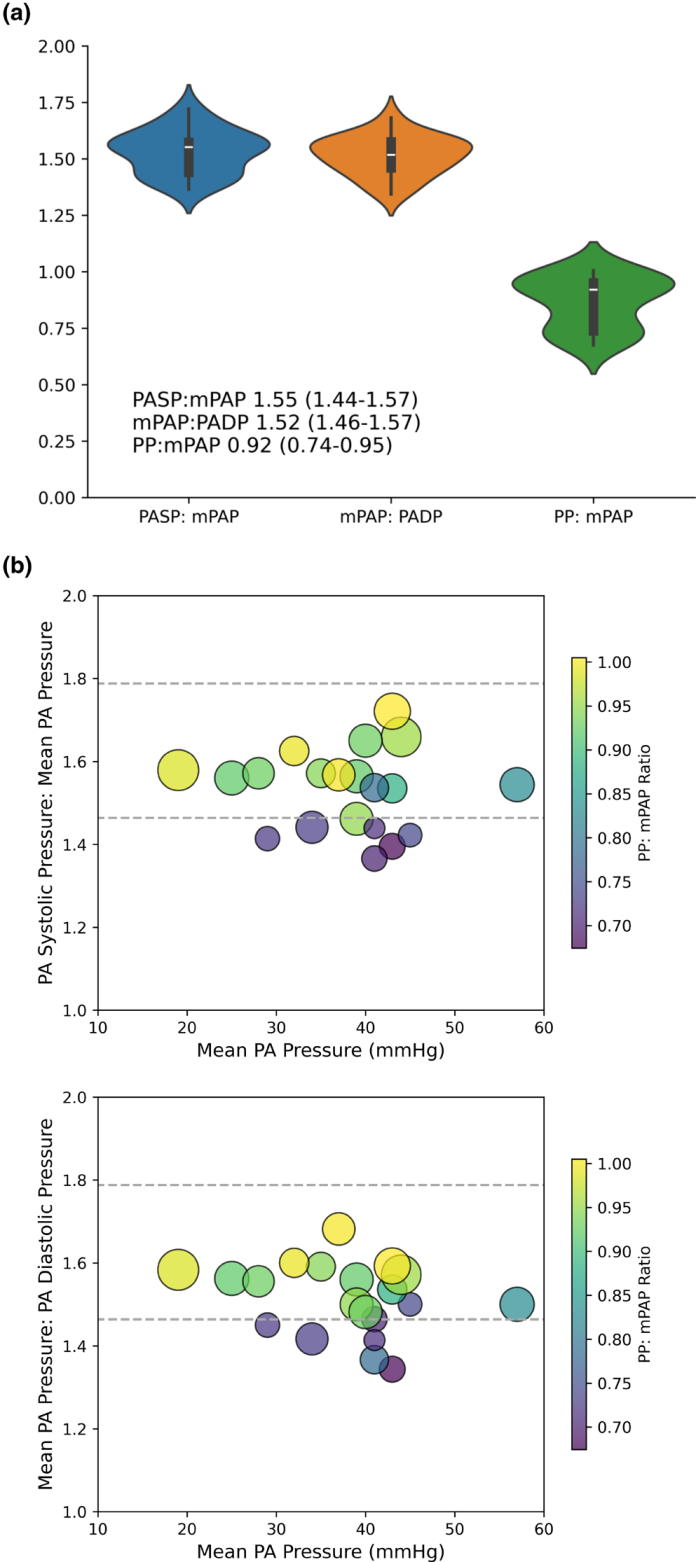
(a) Violin plots of the PASP: mPAP, mPAP: PADP, and PP: mPAP ratios in 20 patients with advanced HF from high‐fidelity pressure recordings. The PASP: mPAP and mPAP: PADP ratios within the defined range of 1.464–1.788 were considered consistent with the Φ hypothesis. (b) Scatter plots of the PASP: mPAP and mPAP: PADP ratios over the range of mPAP. Lower PASP: mPAP and mPAP: PADP ratios were associated with a lower PP: mPAP ratio. The color scale corresponds to the PP:mPAP ratio, and the size of the dots corresponds to stroke volume index.

The characteristics of the 20 patients in the high‐fidelity HF cohort have been reported previously and summarized in Table S2. The median PASP: mPAP and mPAP: PADP ratios were within the bounds that would be consistent with the Φ hypothesis (Figure 2a). The regression coefficients of mPAP‐to‐PASP and PADP‐to‐mPAP‐to‐PADP were 0.65 and 0.70 (Figure S1). However, a bimodal distribution was evident on the violin plots. Eight of the 20 patients with advanced HF had PASP: mPAP and/or mPAP: PADP ratios below the lower limit (Figure 2b), which was associated with significantly lower PP: mPAP ratio (0.72 (0.71–0.73) vs. 0.94 (0.92–0.99), *p* = 0.002). The PP: mPAP ratio remained within the range defined for patients with HF.

The PP: mPAP in patients with advanced HF significantly correlated inversely with right atrial pressure, PA wedge pressure, and heart rate, and directly with SV (Figure S2). The eight patients with low PASP: mPAP and/or mPAP: PADP ratios had higher right atrial pressure (17 (16–19) vs. 12 (10–13) mmHg, *p* = 0.002) and heart rate (88 (79–96) vs. 70 (64–74), *p* = 0.012) and lower SV index (18 (16–23) vs. 30 (26–33) mL, *p* = 0.002). The difference in PA wedge pressure was not significant (28 (25–28) vs. 22 (19–26), *p* = 0.069).

### 3.2 Cardiogenic shock cohort

The demographics and hemodynamic data of the patients with CS are shown in Table 1. The majority of the patients had CS due to end‐stage HF, were deteriorating on vasoactive drugs (60/93 or 65% were Society of Cardiovascular Angiography and Interventions (SCAI) stage D). Dobutamine, milrinone, and/or dopamine were used in all cases with the addition of epinephrine and norepinephrine in 22% and 89%, respectively.

**TABLE 1:** Patient characteristics and hemodynamic data (*n* = 93).

Parameter	All	Normal ratio	Low ratio
	(*n* = 93)	(*n* = 76)	(*n* = 17)
Age >50 years (*n*, %)	51 (55%)	42 (55%)	9 (53%)
Males (*n*, %)	67 (72)	51 (76)	16 (94)*
BMI (kg/m^2^)	26.9 ± 3.5	26.5 ± 2.8	27.0 ± 3.7
Etiology (*n*, %)			
Acute myocardial infarction	23 (25)	19 (25)	4 (24)
End‐stage heart failure			
Ischemic	38 (40)	33 (43)	9 (52)
Non‐ischemic	23 (25)	19 (25)	3 (18)
Other	9 (10)	5 (7)	1 (6)
NT‐pro BNP (pg/mL)	5032 (3019–9125)	6199 (3806–8440)	4660 (2894–9144)
Lactate (mmol/L)	5.3 (4.0–6.3)	4.6 (3.8–6.4)	5.6 (4.6–6.3)
BE	−5.8 (−2.8 to −7.8)	−5.7 (−2.6 to −8.5)	−5.9 (−2.8 to −7.8)
LVEF (%)	15 (10–20)	10 (10–20)	18 (10–20)
Epinephrine (*n*, %)	20 (22)	16 (21)	4 (24)
Norepinephrine (*n*, %)	83 (89)	68 (89)	15 (88)
Vasoactive inotrope score	12 (8–16)	11 (8–15)	14 (10–16)
Systolic BP (mmHg)	85 ± 6	85 ± 6	85 ± 6
Diastolic BP (mmHg)	54 ± 5	54 ± 5	53 ± 5
Mean arterial BP (mmHg)	67 ± 5	67 ± 4	67 ± 5
Heart rate (BPM)	95 ± 13	93 ± 12	105 ± 11*
Right atrial pressure (mmHg)	17 ± 6	16 ± 5	22 ± 4*
PA systolic pressure (mmHg)	57 (49–64)	59 (50–65)	50 (47–62)
PA diastolic pressure (mmHg)	28 (24–31)	27 (23–31)	30 (29–35)*
Mean PA pressure (mmHg)	39 (35–44)	39 (34–44)	38 (37–45)
PA wedge pressure (mmHg)	27 ± 5	26 ± 5	29 ± 4*
Cardiac index (L/min/m^2^)	1.64 (1.41–1.78)	1.66 (1.55–1.80)	1.44 (1.21–1.62)*
Pulmonary vascular resistance (WU)	4.2 ± 0.9	4.2 ± 1.0	4.2 ± 0.9
Cardiac power output index (W/m^2^)	0.20 (0.17–0.25)	0.19 (0.16–0.21)	0.15 (0.12–0.18)*
PA systolic: mean PA pressure	1.44 ± 0.12	1.47 ± 0.10	1.30 ± 0.06*
Mean PA: PA diastolic pressure	1.42 ± 0.09	1.44 ± 0.07	1.30 ± 0.04*
PA pulse pressure: Mean PA pressure	0.71 (0.64–0.83)	0.74 (0.68–0.87)	0.53 (0.48–0.57)*

*Note*: Data shown as mean ± standard deviation, median (interquartile range) or absolute numbers (percentage).

Abbreviations: BE, base excess; BMI, body mass index; BP, blood pressure; LVEF, left ventricular ejection fraction; NT‐proBNP, N‐terminal B‐natriuretic peptide; PA, pulmonary artery.

* *p* < 0.05.

**FIGURE 3:**
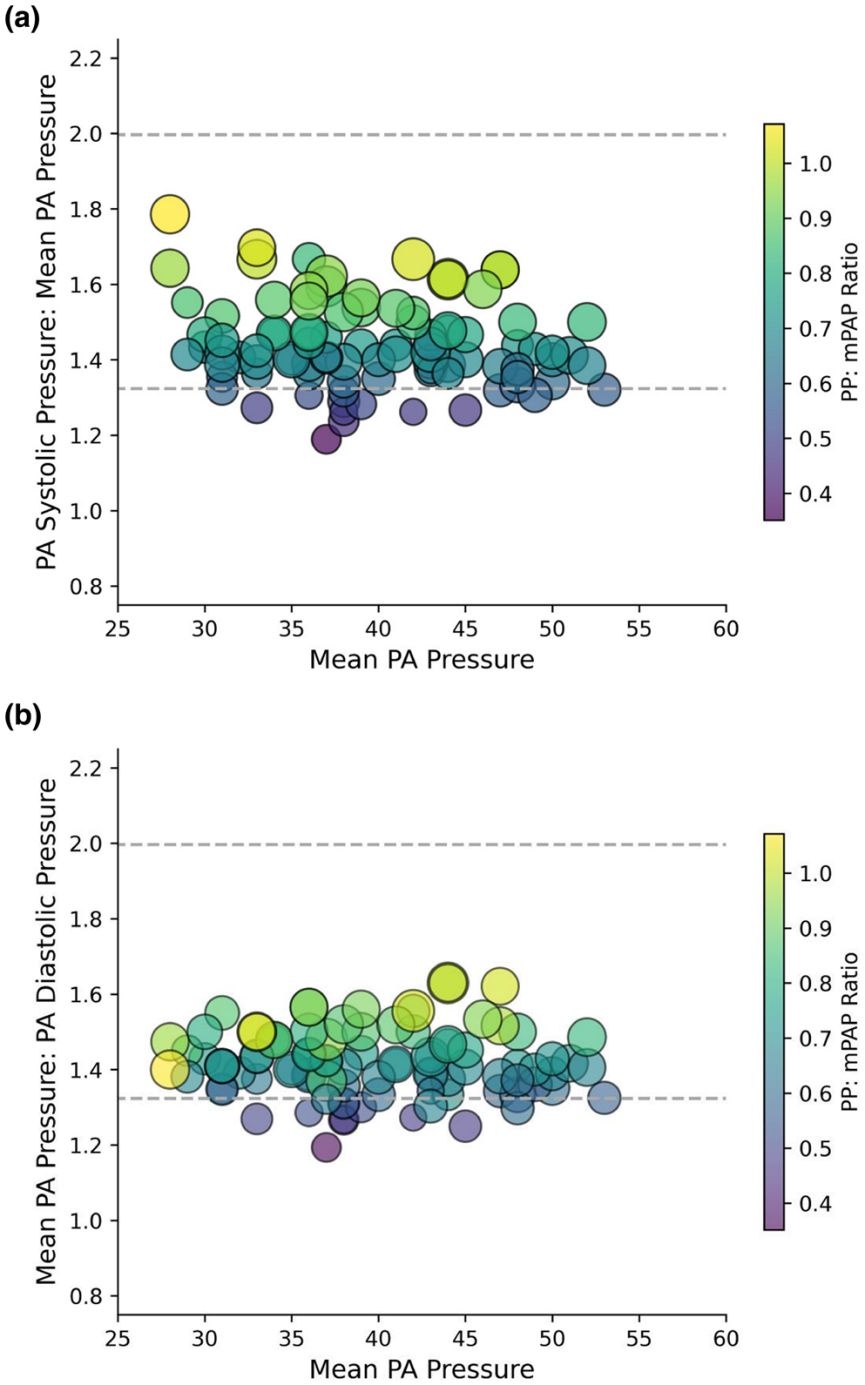
(a) Scatter plot of PASP: mPAP ratios over the range of mPAP in the cardiogenic shock cohort. (b) Scatter plot of mPAP: PADP ratios over the range of mPAP in the cardiogenic shock cohort. In both figures, accounting for a 10% error in individual pressure measurements, ratios within the range of 0.82–1.22 Φ (dashed lines) were considered consistent with the Φ hypothesis. The size and color of the markers are related to the PP: mPAP ratio. Lower PASP: mPAP and mPAP: PADP ratios were associated with lower PP: mPAP ratio.

The median mPAP was 39 (35–44) mmHg. PP correlated with SV index (*R* ^2^ = 0.684, *p* < 0.001), but the correlation between SV and mPAP was not significant (*R* ^2^ = 0.115, *p* = 0.170). The median PASP: mPAP of 1.42 was 88% of Φ and the median mPAP: PADP of 1.41 was 87% of Φ. Based on the 10% error in the PA pressure measurements, 76 patients (82%) had PASP: mPAP and mPAP: PADP ratios that were consistent with the Φ hypothesis (Cross‐tabulation shown in Figure S3). Seventeen patients (18%) had PASP: mPAP and mPAP: PADP ratios below the 10% error margin—“low ratio group” (Figure 3a,b). The difference between the PASP: mPAP and mPAP: PADP ratios were not statistically significant in both the “low ratio group” and the “normal” ratio group (i.e., the ratios of PASP: mPAP ≈ mPAP: PADP).

The “low ratio group” had significantly lower PP: mPAP ratio (0.53 (0.48–0.57) vs. 0.74 (0.68–0.87), *p* < 0.001) that was below the lower for patients with HF, as defined by previous studies. The lower PP: mPAP ratio in the “low ratio group” was the result of significantly higher PADP (30 (29–35) vs. 27 (23–31) mmHg, *p* = 0.003), with no significant difference in PASP and mPAP (50 (47–62) vs. 59 (50–65) mmHg, *p* = 0.073; 38 (37–45) vs. 39 (34–44) mmHg, *p* = 0.426, respectively).

**FIGURE 4:**
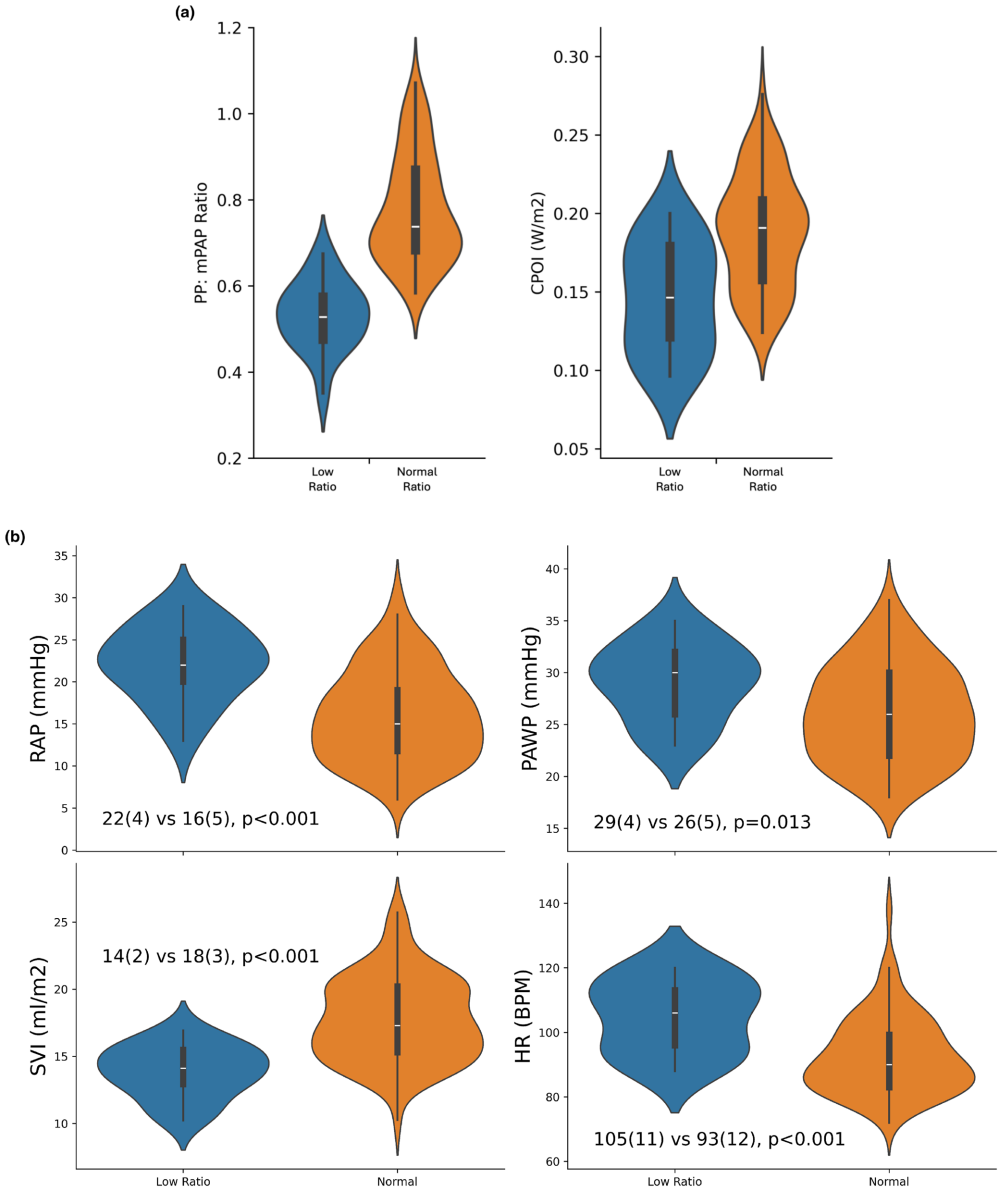
(a) Patients in the “low ratio group” (i.e., PASP: mPAP and mPAP: PADP ratios below the 10% error margin) had significantly lower PP: mPAP ratio and CPOI. (b) The lower CPOI in the “low ratio group” is related to higher right atrial pressure and lower stroke volume index and CI, despite the higher heart rate.

The lower PP: mPAP was associated with lower CPOI (0.15 (0.12–0.18) vs. 0.19 (0.16–0.21), *p* < 0.001) (Figure 4a). The PP: mPAP ratio was significantly and directly correlated with SV index and inversely with heart rate and PAWP, but not mean arterial blood pressure or other parameters such as NT‐proBNP and lactate level (Figures S4a,b and S5). The lower CPOI in the “low ratio group” was the result of higher RAP, lower SV index, and lower CI (1.44 (1.21–1.62) vs. 1.66 (1.55–1.80) L/min/m^2^, *p* = 0.003), despite the higher heart rate, as MAP was comparable between groups (67 ± 4 vs. 67 ± 5, *p* = 0.903) (Figure 4b). There was no significant difference in pulmonary vascular resistance, PVR (4.2 ± 1.0 vs. 4.2 ± 0.9 WU, *p* = 0.919), pulmonary artery capacitance, PAC (1.27 ± 0.20 vs. 1.18 ± 0.19 mL/mmHg, *p* = 0.192) and diastolic time constant, tau (0.31 ± 0.07 vs. 0.29 ± 0.04 s, *p* = 0.281).

## 4 DISCUSSION

In this study, we tested the Φ hypothesis in the pulmonary circulation of patients with HF and CS. We were not able to refute the Φ hypothesis in the pulmonary circulation in most patients with advanced HF and CS. However, the pulmonary circulation deviated from the Φ hypothesis in patients with advanced HF and CS with more severe hemodynamic compromise.

Chemla et al. (2019) described proportionality between PASP and mPAP under different conditions, which formed the basis for the Φ hypothesis. However, the Φ hypothesis has not been specifically evaluated in patients with advanced HF and CS. To fulfill the Φ hypothesis, the PASP: mPAP ratio should approximate the mPAP: PADP ratio and both ratios should approximate Φ, or 1.618. Our data suggest that the Φ hypothesis could not be refuted in most patients with advanced HF and CS based on these criteria. However, there was notable heterogeneity within the patient groups. A significant minority of patients with advanced HF (8/20) and CS (17/93) did not meet the criteria for the Φ hypothesis because both PASP: mPAP and mPAP: PADP ratios were below the defined limits (i.e., PASP: mPAP ratio ≈ mPAP: PADP ratio, but < Φ or 1.618).

The finding that the PP did not approximate mPAP (i.e., PP: mPAP <1.0) also implied that the pulmonary circulation deviated from the Φ hypothesis in these patients with “low ratios”. Such deviation from the Φ hypothesis in the pulmonary circulation was associated with more severe hemodynamic compromise in these patients with HF and CS. Of note, broader limits were adopted in the cohort of patients with CS to account for greater measurement error with fluid‐filled systems used, which precluded direct comparisons with the high‐fidelity measurements in the advanced HF cohort.

We specifically examined the PP: mPAP ratio as this was a criterion for the Φ hypothesis. In the absence of published data for a ‘normal’ range of PP: mPAP ratio in HF, we reviewed 10 studies of patients with HF and PHLHD, which yielded a median PP: mPAP ratio of 0.89 (0.79–0.96). This range of PP: mPAP was comparable to other studies of patients with PH that were considered consistent with the Φ hypothesis (Chemla et al., 2019). The PP: mPAP ratio was slightly lower in our advanced HF cohort with high‐fidelity pressure measurements but was nonetheless within the defined limits. However, in patients with CS, particularly in the “low ratio group”, the PP: mPAP ratio breached the lower limits, which is consistent with a deviation from the Φ hypothesis in the “low ratio group” of patients with CS.

The relationship between PP: mPAP ratio and other hemodynamic parameters may be of interest. For example, with a strong correlation with SV index, PP: mPAP ratio may be a useful surrogate for SV index. The change in PP: mPAP ratio with SV may be explained by the mathematical determinants of both PP and mPAP. Based on the simplified equation for PAC (PP = SV/PAC), PP is directly related to SV and inversely related to PAC (which is affected by PAWP). As such, it is not surprising that PP correlated with SV in this study. This finding is also consistent with our previous observation that changes in PP tracked changes in SV (Yim, Drury, & Lim, 2024).

This simplified PAC equation indicates that PP changes with SV by the coefficient of 1/PAC. For a PAC of 1.2 mL/mmHg, PP will change by 0.83 mmHg for each mL change in SV. The reduction in PP with reduction in SV may manifest as an increase in PADP because of higher downstream pressures (higher PAWP) and a faster heart rate. At any level of diastolic time constant (tau = product of resistance and compliance), a faster heart rate is associated with higher diastolic blood pressure.

Stroke volume is a more modest determinant of mPAP. Rearranging the PVR equation, the coefficient is the product of PVR and HR (or the ratio of PVR‐to‐cardiac interval, *t*), with the y‐intercept determined by PAWP (assuming West zone 3 conditions):

$$\text{mPAP}=\left(\mathrm{PVR}\times \mathrm{HR}\right)\times \mathrm{SV}+\text{PAWP}$$

$$\text{mPAP}=\frac{\mathrm{PVR}}{t}\times \mathrm{SV}+\text{PAWP}$$

This equation indicates that a faster heart rate (i.e., shorter cardiac interval, t) will mitigate against the reduction in mPAP from the reduction in SV. This may explain the poor correlation between SV and mPAP. Based on a PVR of 4.2WU (or 0.252 mmHg/mL.s) in the CS cohort in this study, and simplifying the heart rate to 100 bpm (i.e., cardiac interval of 600 ms), the mPAP will change by PVR/t or 0.42 mmHg per mL change in SV. At high levels of PAWP of 29 mmHg, the change in mPAP will be relatively modest [Graphical Abstract]. The steeper reduction in PP relative to mPAP, especially in the context of high PAWP, may explain the progressive diminution of the PP:mPAP ratio with deteriorating hemodynamic status in our cohort of patients with CS.

Although PVR is often regarded as the measure of right ventricular (RV) afterload, elevation in PAWP imposes significant pulsatile load on the RV (Saouti, Westerhof, Postmus, & Vonk‐Noordegraaf, 2010). The RV must increase contractility and/or recruit additional preload (i.e., increase end‐diastolic volume) in the face of increased afterload to maintain SV. The diminution of SV at increased afterload thus reflects the exhaustion of contractile and preload reserves, leading to RV failure due to afterload mismatch. The significantly higher right atrial pressure in the ‘low ratio’ group is consistent with more severe RV dysfunction. The mathematical model suggests that in the setting of elevated PAWP, low pulmonary artery capacitance and tachycardia, reduction in SV will reduce PP to a greater extent than the reduction in mPAP, thereby reducing the PP: mPAP ratio. Indeed, our findings are consistent with previous studies that showed that both higher PAWP (Tedford et al., 2012) and faster HR (Metkus et al., 2016) reduced Tau and would violate the assumption of proportionality of pulmonary artery pressures. In this regard, deviation from Φ and the reduction in PP: mPAP ratio may reflect afterload mismatch and the uncoupling of the RV‐PA system due to RV failure. It would be of interest to explore if deviation from the golden ratio is associated with higher mortality, and if restoration of the golden ratio is associated with improved outcomes in patients with CS.

This coupling between the RV and PA can also be viewed through the relationship between the cardiac interval, t and the diastolic time constant, Tau. Based on the arterial Windkessel, Tau is the exponential decay in diastolic pressure after one “unit of time”, the response of which is e^−1^, or 0.36788, or time to 63.2% of the final value. As the product of PVR and PAC, Tau can be re‐written as:

$$\mathrm{Tau}=\frac{\left(\text{mPAP}-\text{PAWP}\right)}{\mathrm{HR}\times \mathrm{SV}}\times \mathrm{PAC}$$

Rearranging the equation with cardiac interval, *t* = 1/HR:

$$\frac{\mathrm{Tau}}{t}=\frac{\left(\text{mPAP}-\text{PAWP}\right)}{\mathrm{SV}}\times \mathrm{PAC}$$

As transpulmonary gradient, TPG = mPAP‐PAWP, the equation can be rewritten as:

$$\frac{t}{\mathrm{Tau}}=\frac{\mathrm{SV}}{\mathrm{TPG}\times \mathrm{PAC}}$$

This dimensionless ratio of *t*:Tau describes the SV at any level of afterload (lumping together the Ohmic pressure gradient and pulsatile load)—the lower the ratio, the smaller the SV at any level of afterload, implying greater the afterload mismatch. Both *t*/Tau and SV/(TPGxPAC) were significantly lower in the “low ratio” compared to the “normal ratio” group (1.94 ± 0.39 vs. 2.34 ± 0.41 and 1.92 ± 0.38 vs. 2.34 ± 0.42 both *p* < 0.001) because of the faster HR at comparable Tau in the former.

### 4.1 Study limitations

This study has several limitations. Firstly, there are inherent biases associated with observational studies, especially as this study included only a relatively small cohort of patients from a single center. We avoided direct comparisons between patients with advanced HF and CS due to the different measurement modalities. Secondly, we used the limits for PASP: mPAP and mPAP: PADP ratios that were defined by Chemla et al. (2019), which were developed for patients with PH. Thirdly, this study did not examine the effect of therapeutic interventions on PASP: mPAP, mPAP: PADP, and PP: mPAP ratios. Future study should examine if improvement in systemic hemodynamic parameters (e.g., with inotropes or mechanical circulatory support) is accompanied by realignment with the Φ hypothesis in the pulmonary circulation. Fourthly, this study has not explored the prognostic significance of the Φ hypothesis. Based on previous studies in CS, the lower CPOI would be expected to be associated with higher short‐term mortality.

## 5 CONCLUSION

In summary, our data failed to refute the Φ hypothesis in the pulmonary circulation in the majority of patients with advanced HF and CS. However, the pulmonary circulation of patients with more severe hemodynamic compromise deviated from the Φ hypothesis, possibly reflecting RV‐PA uncoupling due to afterload mismatch. Deviation from the Φ hypothesis and low PP: mPAP ratio may identify a group of patients with HF and CS with more severe circulatory failure.

## FUNDING INFOMATION

This work was supported by the Heart Research UK (NET31/19).

## CONFLICT OF INTEREST STATEMENT

No conflicts of interest to declare.

## ETHICS STATEMENT

This study has been approved by the Health Research Authority and Health Care Research Wales ethics committee (reference 20/WM/0022) with written informed consent from participants.

## Supporting information


Figures S1–S5.

Tables S1–S2.
