# Supplementary material for: The golden ratio in the pulmonary circulation in patients with heart failure and cardiogenic shock
Source: Physiol Rep. 2025 Mar 28;13(7):e70287. doi: 10.14814/phy2.70287 (PMC11953056; doi:10.14814/phy2.70287)

**The Golden Ratio in the Pulmonary Circulation in Patients with Heart Failure and Cardiogenic Shock - Supplementary Data**

Supplementary Figure 1: Proportional relationship between PASP, mPAP and PADP. The coefficients of 0.65 and 0.70 approximate the Golden Ratio.


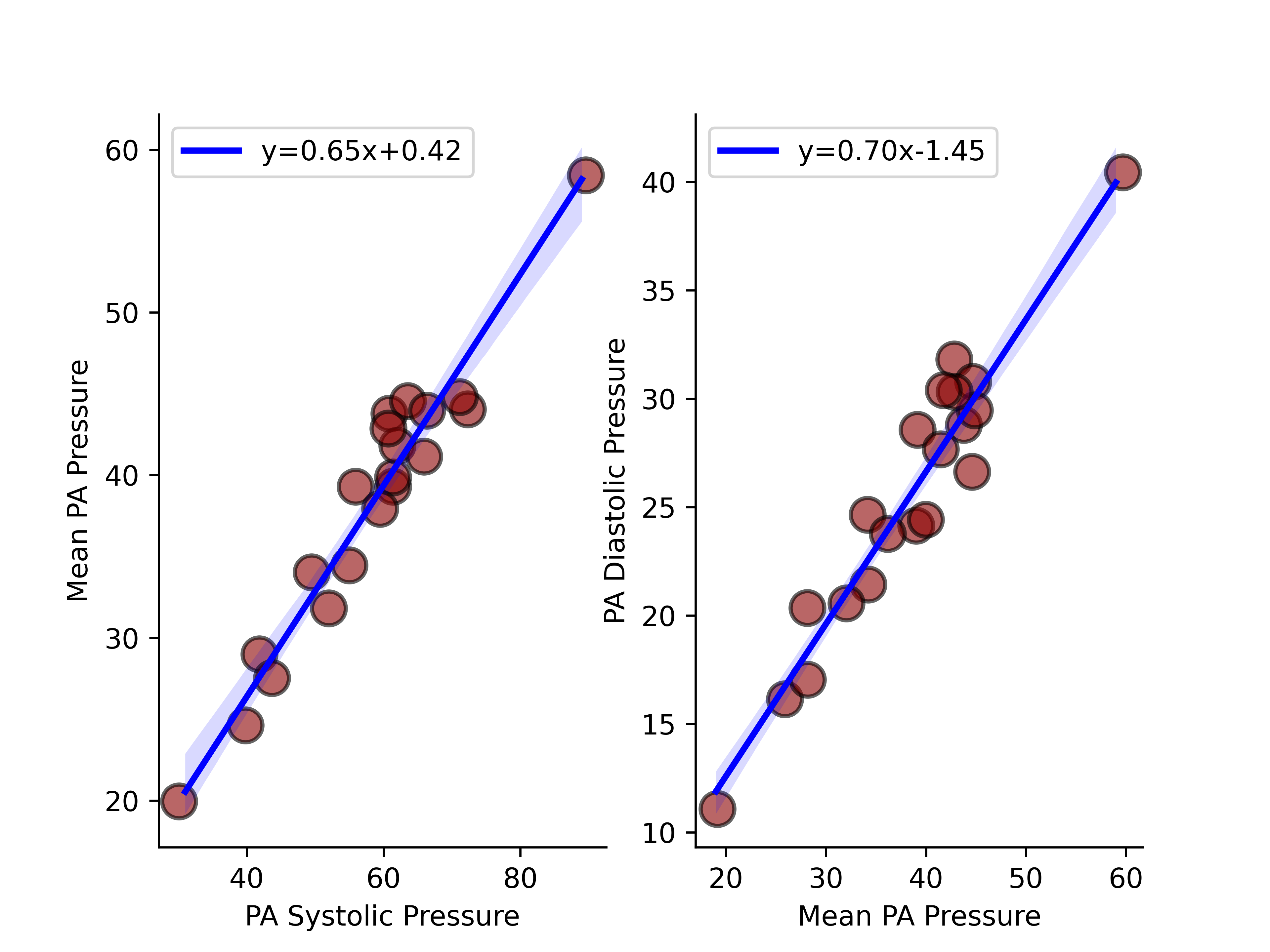


Supplementary Figure 2: Correlation between PP: mPAP ratio and other hemodynamic parameters in patients with advanced HF.


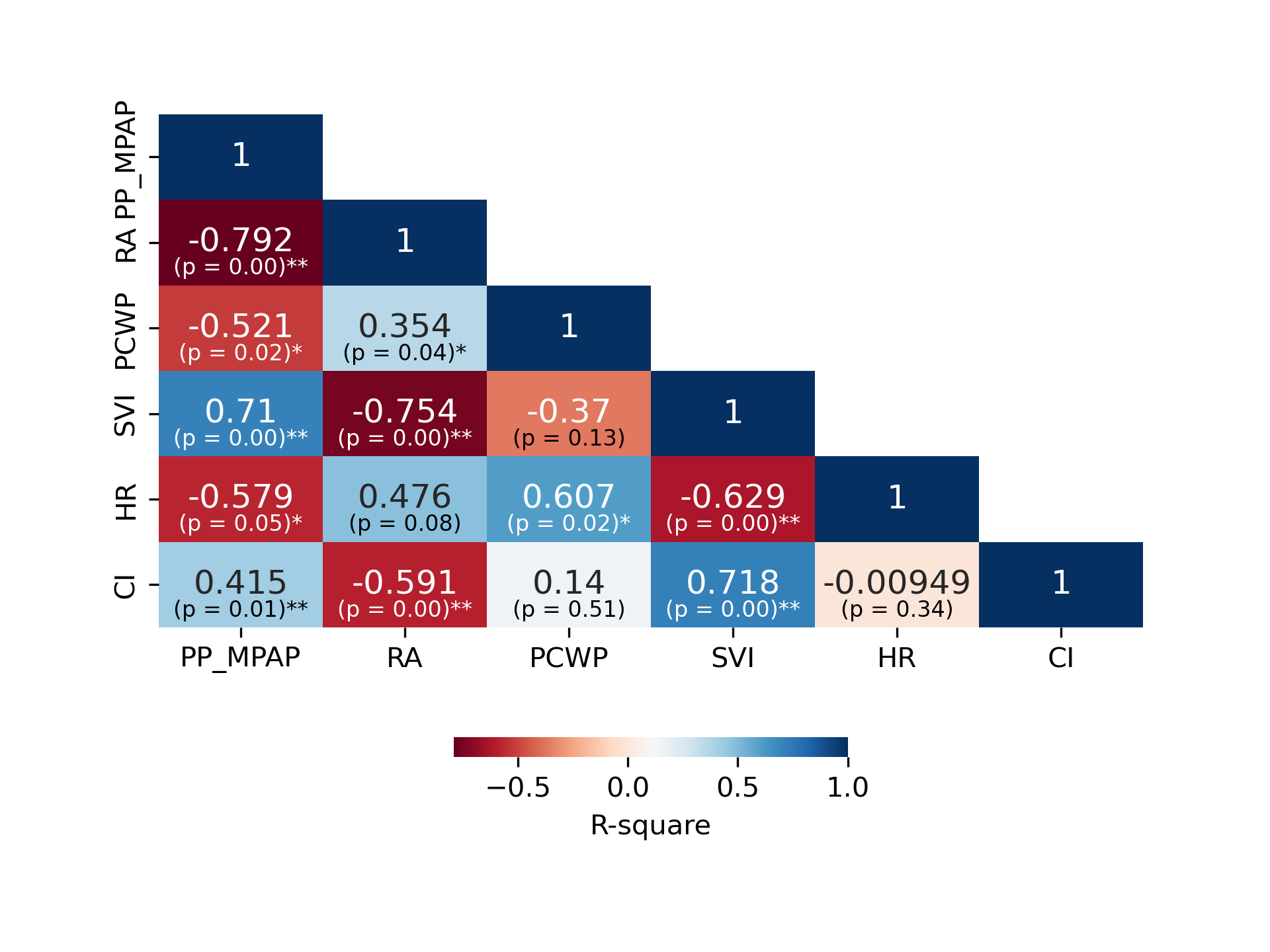


Supplementary Figure 3: Low ratio group consists of the 3 groups in red, i.e. low PASP: mPAP and/or low mPAP: PADP ratios.


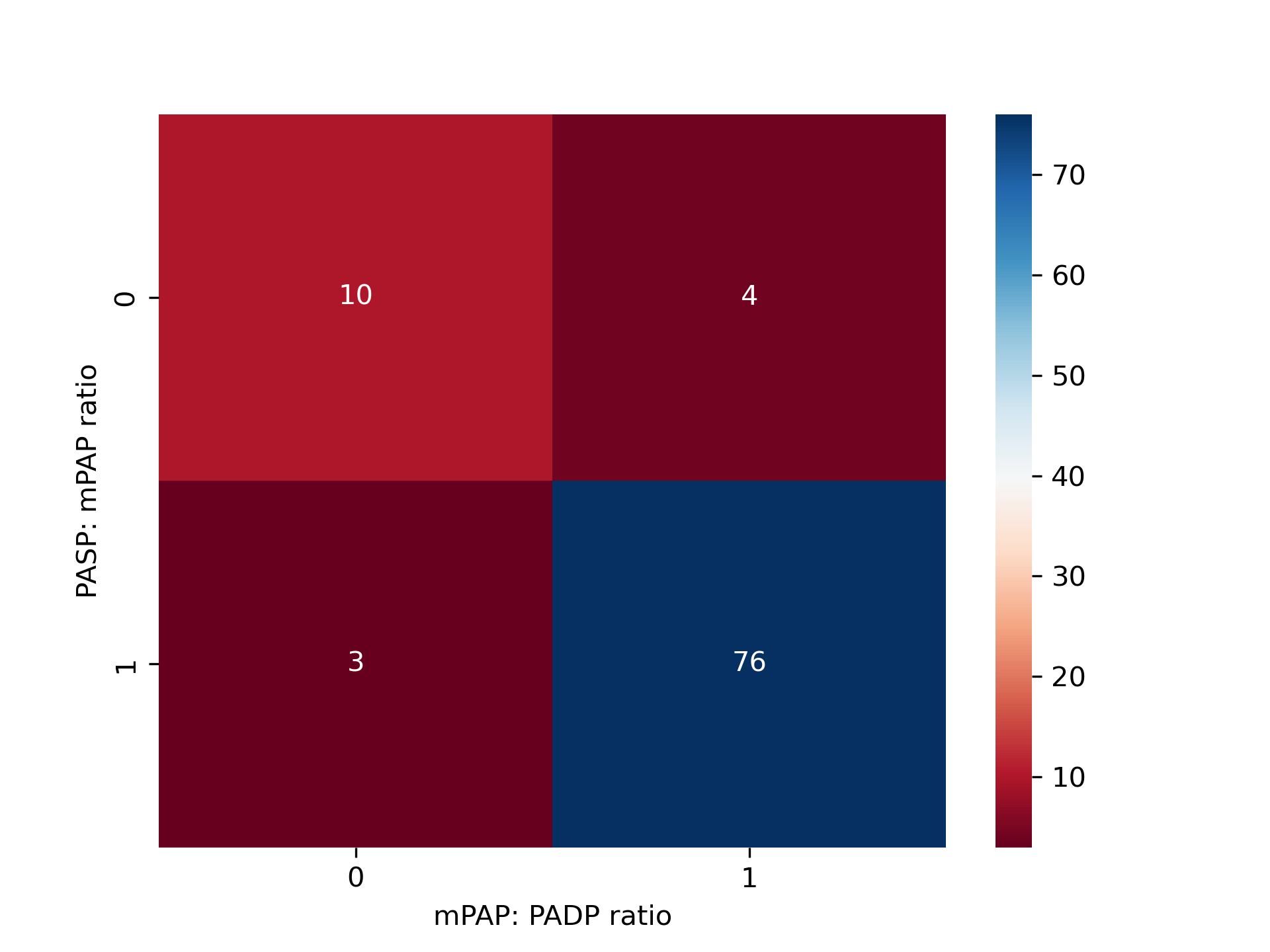


Supplementary Figure 4a: Correlation between PP: mPAP ratio and hemodynamic parameters


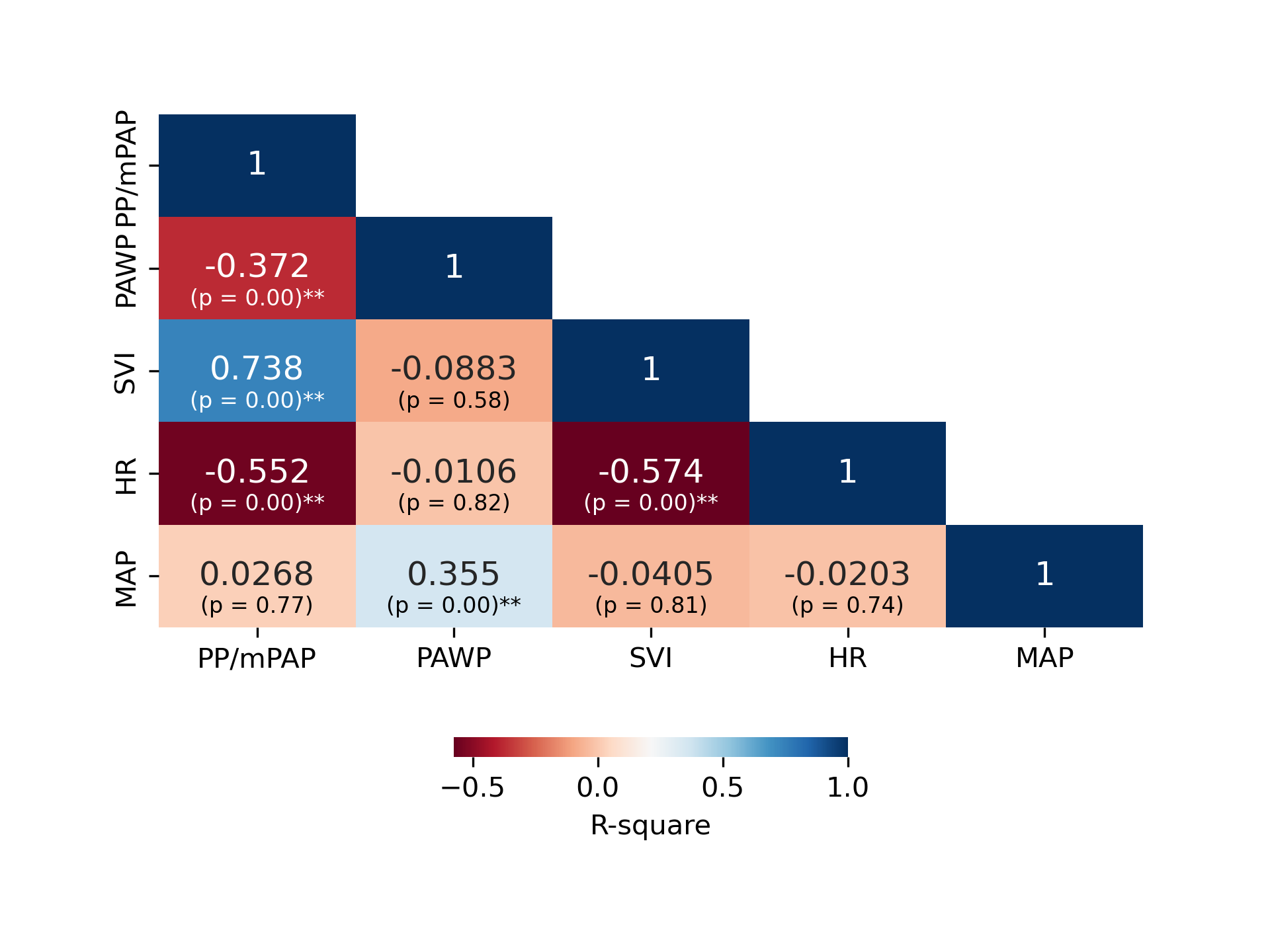


Supplementary Figure 4b: Correlation between PP: mPAP ratio and non-hemodynamic parameters


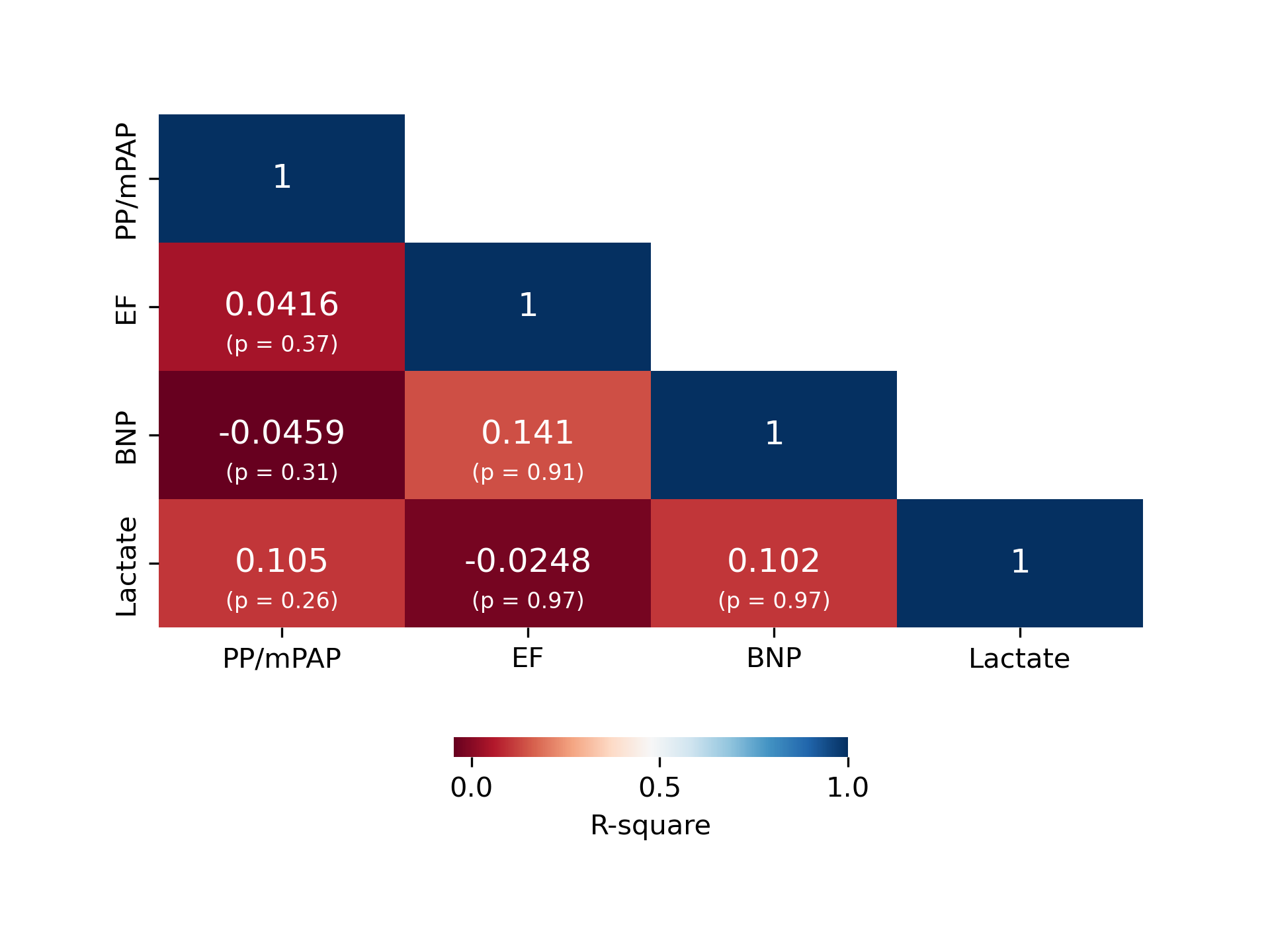


Supplementary Figure 5: CPOI is significantly correlated with PP:mPAP ratio (R^2^=0.359, p<0.001)


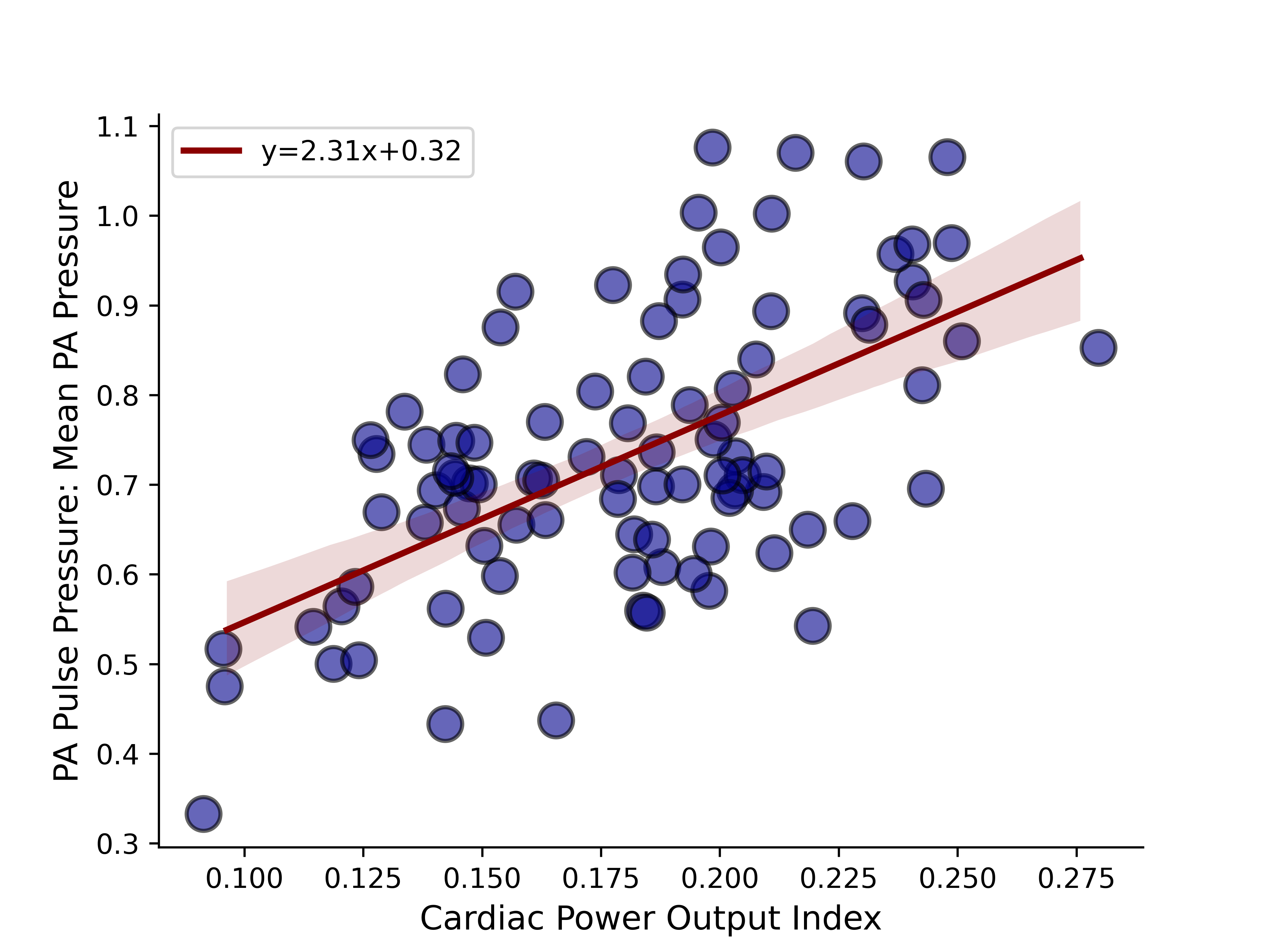

Supplement: Supplementary file 1 — Figures S1–S5. [file PHY2-13-e70287-s002.docx]
