# Supplementary material for: The golden ratio in the pulmonary circulation in patients with heart failure and cardiogenic shock
Source: Physiol Rep. 2025 Mar 28;13(7):e70287. doi: 10.14814/phy2.70287 (PMC11953056; doi:10.14814/phy2.70287)
Supplement: Supplementary file 2 — Tables S1–S2. [file PHY2-13-e70287-s001.docx]

**The Golden Ratio in the Pulmonary Circulation in Patients with Heart Failure and Cardiogenic Shock - Supplementary Data**

Supplementary Table 1: Studies of pulmonary hypertension due to left heart disease. The median PP: mPAP ratio was 0.893 (0.788-0.964), with the lowest reported ratio of 0.700.

| **Study** | **PASP** | **PADP** | **PP** | **mPAP** | **PP: mPAP** | **PAWP** | **CO/CI** | **HR** | **SV/SVI** |
| --- | --- | --- | --- | --- | --- | --- | --- | --- | --- |
| Wright et al^[[1]](#endnote-1)^  PHLHD (n=72)  PAH (n=9)  No PH (n=60) | 48  46  28 | 22  18  11 | 26  28  17 | 33  29  19 | 0.788  0.966  0.895 | 21  13  10 | 1.8  1.8  2 | 73  74  69 | 25  24  29 |
| Gerges et al^[[2]](#endnote-2)^  Pre-capillary (n=137)  Passive (n=604)  DPG<7mmHg (n=311)  DPG>=7 (n=179) | 78  50  61  68 | 31  23  25  33 | 47  27  36  35 | 48  34  40  45 | 0.979  0.794  0.900  0.778 | 10  25  23  21 | 2.6  2.6  2.6  2.6 | 80  76  78  82 |  |
| Miller et al^[[3]](#endnote-3)^  Passive PH (n=151)  Mixed PH (n=186) | 48  62 | 23  31 | 25  31 | 34  42 | 0.735  0.738 | 24  25 | (CO)  4.8  3.7 | 78  77 | (SV)  64  50 |
| Al-Naamani et al^[[4]](#endnote-4)^  IPC (n=47)  CPC (n=26) |  |  | 38  35 | 41  35 | 0.927  1.000 | 21  21 | (CO)  4.7  4.9 | 71  71 | (SV)  69  74 |
| Palazzini et al^[[5]](#endnote-5)^  IPC (n=108)  Intermediate (n=102)  CPC (n=66) | 47  67  81 | 18  22  33 | 29  45  48 | 30  40  52 | 0.967  1.125  0.923 | 20  20  20 | 2.9  2.4  2.4 | 71  71  80 |  |
| Dragu et al^[[6]](#endnote-6)^  No PH (n=129)  Passive (n=124)  Reactive (n=140) | 30  48  70 | 13  22  30 | 18  26  40 | 19  33  45 | 0.947  0.788  0.889 | 11  24  25 |  |  | (SV)  69  67  59 |
| Tampakakis et al^[[7]](#endnote-7)^  DPG ≥7 (n=62)  DPG<7 (n=407) | 60  52 | 32  26 | 28  26 | 40  34 | 0.700  0.765 | 22  26 | 1.9  1.93 | 94  93 |  |
| O’Sullivan et al^[[8]](#endnote-8)^  No PH (n=108)  Pre-cap (n=56)  IPC (n=220)  CPC (n=49) | 32  52  56  74 | 11  20  22  34 | 21  32  34  40 | 19  33  36  50 | 1.105  0.970  0.944  0.800 | 17  12  26  22 | 2.3  2.0  2.1  1.9 | 75  81  78  90 | 31  26  28  22 |
| Lim et al^[[9]](#endnote-9)^  Responders (n=38)  Non-responders (n=60) | 77  66 | 36  32 | 41  34 | 46  42 | 0.891  0.810 | 27  28 | (CO)  3.4  3.7 | 83  79 | (SV)  41  47 |
| Assad et al^[[10]](#endnote-10)^  PAH (n=564)  IPC (n=1456)  CPC (n=364) | 72  53  69 | 29  23  34 | 43  30  35 | 45  36  47 | 0.956  0.833  0.745 | 9  24  22 | 2.5  2.7  2.5 | 78  76  82 | (SV)  60  71  64 |

Supplementary Table 2: Characteristics of patients with advanced HF (Yim IHW, Parker KH, Drury NE, Lim HS. Pulmonary artery wave intensity analysis in pulmonary hypertension associated with heart failure and reduced left ventricular ejection fraction. Pulm Circ. 2024 Feb 12;14(1):e12345.)

| Age (years) | 51 ± 11 |
| --- | --- |
| Males (%) | 75% |
| BMI (kg/m^2^) | 28 ± 5 |
| Sodium (mmol/l) | 137 ± 4 |
| Creatinine (umol/l) | 107 (82-137) |
| Bilirubin (umol/l) | 17.5 (10-40) |
| NTproBNP (pg/ml) | 6764 ± 4113 |
| LVEF (%) | 21 (11-25) |
| RA pressure (mmHg) | 14 ± 4 |
| PASP (mmHg) | 59 (51-65) |
| PADP (mmHg) | 27 (22-28) |
| mPAP (mmHg) | 40 (34-43) |
| PAWP (mmHg) | 25 (20-27) |
| Heart rate (BPM) | 74 (70-86) |
| Cardiac index (L/min/m^2^) | 1.92 (1.67-2.19) |

BMI: body mass index; LVEF: left ventricular ejection fraction; RA: right atrial; PASP: pulmonary artery systolic pressure; PADP: pulmonary artery diastolic pressure; mPAP: mean pulmonary artery pressure; PAWP: pulmonary artery wedge pressure

References

1. Wright SP, Moayedi Y, Foroutan F, Agarwal S, Paradero G, Alba AC, Baumwol J, Mak S. Diastolic Pressure Difference to Classify Pulmonary Hypertension in the Assessment of Heart Transplant Candidates. Circ Heart Fail. 2017 Sep;10(9):e004077. [↑](#endnote-ref-1)
2. Gerges C, Gerges M, Lang MB, Zhang Y, Jakowitsch J, Probst P, Maurer G, Lang IM. Diastolic pulmonary vascular pressure gradient: a predictor of prognosis in "out-of-proportion" pulmonary hypertension. Chest. 2013 Mar;143(3):758-766. [↑](#endnote-ref-2)
3. Miller WL, Grill DE, Borlaug BA. Clinical features, hemodynamics, and outcomes of pulmonary hypertension due to chronic heart failure with reduced ejection fraction: pulmonary hypertension and heart failure. JACC Heart Fail. 2013 Aug;1(4):290-299. [↑](#endnote-ref-3)
4. Al-Naamani N, Preston IR, Paulus JK, Hill NS, Roberts KE. Pulmonary Arterial Capacitance Is an Important Predictor of Mortality in Heart Failure With a Preserved Ejection Fraction. JACC Heart Fail. 2015 Jun;3(6):467-474 [↑](#endnote-ref-4)
5. Palazzini M, Dardi F, Manes A, Bacchi Reggiani ML, Gotti E, Rinaldi A, Albini A, Monti E, Galiè N. Pulmonary hypertension due to left heart disease: analysis of survival according to the haemodynamic classification of the 2015 ESC/ERS guidelines and insights for future changes. Eur J Heart Fail. 2018 Feb;20(2):248-255. [↑](#endnote-ref-5)
6. Dragu R, Rispler S, Habib M, Sholy H, Hammerman H, Galie N, Aronson D. Pulmonary arterial capacitance in patients with heart failure and reactive pulmonary hypertension. Eur J Heart Fail. 2015 Jan;17(1):74-80. [↑](#endnote-ref-6)
7. Tampakakis E, Leary PJ, Selby VN, De Marco T, Cappola TP, Felker GM, Russell SD, Kasper EK, Tedford RJ. The diastolic pulmonary gradient does not predict survival in patients with pulmonary hypertension due to left heart disease. JACC Heart Fail. 2015 Jan;3(1):9-16 [↑](#endnote-ref-7)
8. O'Sullivan CJ, Wenaweser P, Ceylan O, Rat-Wirtzler J, Stortecky S, Heg D, Spitzer E, Zanchin T, Praz F, Tüller D, Huber C, Pilgrim T, Nietlispach F, Khattab AA, Carrel T, Meier B, Windecker S, Buellesfeld L. Effect of Pulmonary Hypertension Hemodynamic Presentation on Clinical Outcomes in Patients With Severe Symptomatic Aortic Valve Stenosis Undergoing Transcatheter Aortic Valve Implantation: Insights From the New Proposed Pulmonary Hypertension Classification. Circ Cardiovasc Interv. 2015 Jul;8(7):e002358.  [↑](#endnote-ref-8)
9. Lim HS, Zaphiriou A. Sodium Nitroprusside in Patients With Mixed Pulmonary Hypertension and Left Heart Disease: Hemodynamic Predictors of Response and Prognostic Implications. J Card Fail. 2016 Feb;22(2):117-24.  [↑](#endnote-ref-9)
10. Assad TR, Hemnes AR, Larkin EK, Glazer AM, Xu M, Wells QS, Farber-Eger EH, Sheng Q, Shyr Y, Harrell FE, Newman JH, Brittain EL. Clinical and Biological Insights Into Combined Post- and Pre-Capillary Pulmonary Hypertension. J Am Coll Cardiol. 2016 Dec 13;68(23):2525-2536. [↑](#endnote-ref-10)
